# PPARγ Modulates Long Chain Fatty Acid Processing in the Intestinal Epithelium

**DOI:** 10.3390/ijms18122559

**Published:** 2017-11-28

**Authors:** Kalina Duszka, Matej Oresic, Cedric Le May, Jürgen König, Walter Wahli

**Affiliations:** 1 Lee Kong Chian School of Medicine, Nanyang Technological University, 11 Mandalay Road, Singapore 308232, Singapore; Kalina.duszka@univie.ac.at; 2 Center for Integrative Genomics, University of Lausanne, Génopode, CH-1015 Lausanne, Switzerland; 3 Department of Nutritional Sciences, University of Vienna, Althanstrasse 14, 1090 Vienna, Austria; juergen.koenig@univie.ac.at; 4 Turku Centre for Biotechnology, University of Turku and Åbo Akademi University, Tykisokatu 6, 20520 Turku, Finland; matej.oresic@utu.fi; 5 Institut du Thorax, INSERM, CNRS, UNIV Nantes, 44007 Nantes, France; cedric.lemay@univ-nantes.fr; 6 Vienna Metabolomics Center (VIME), University of Vienna, Althanstrasse 14, 1090 Vienna, Austria; 7 ToxAlim, Research Center in Food Toxicology, National Institute for Agricultural Research (INRA), 180 Chemin de Tournefeuille, 31300 Toulouse, France

**Keywords:** PPARγ, intestine, lipid metabolism

## Abstract

Nuclear receptor PPARγ affects lipid metabolism in several tissues, but its role in intestinal lipid metabolism has not been explored. As alterations have been observed in the plasma lipid profile of ad libitum fed intestinal epithelium-specific PPARγ knockout mice (iePPARγKO), we submitted these mice to lipid gavage challenges. Within hours after gavage with long chain unsaturated fatty acid (FA)-rich canola oil, the iePPARγKO mice had higher plasma free FA levels and lower gastric inhibitory polypeptide levels than their wild-type (WT) littermates, and altered expression of incretin genes and lipid metabolism-associated genes in the intestinal epithelium. Gavage with the medium chain saturated FA-rich coconut oil did not result in differences between the two genotypes. Furthermore, the iePPARγKO mice did not exhibit defective lipid uptake and stomach emptying; however, their intestinal transit was more rapid than in WT mice. When fed a canola oil-rich diet for 4.5 months, iePPARγKO mice had higher body lean mass than the WT mice. We conclude that intestinal epithelium PPARγ is activated preferentially by long chain unsaturated FAs compared to medium chain saturated FAs. Furthermore, we hypothesize that the iePPARγKO phenotype originates from altered lipid metabolism and release in epithelial cells, as well as changes in intestinal motility.

## 1. Introduction

The digestion of lipids starts in the oral cavity and involves lipases secreted by the lingual glands. The process continues in the stomach, where fats become emulsified and enter the duodenum as fine lipid droplets. There, they are further emulsified, micellized, and processed by bile acids and the pancreatic juice, eventually resulting in the formation of monoglycerides, free glycerol, and free fatty acids (FFAs) [1,2]. CD36 and various Fatty acids biding proteins (FABPs) facilitate long chain fatty acid (LCFA) transport across the apical membrane of enterocytes [3,4]. After entering enterocytes, FFAs and glycerol arrive at the crossroads of several pathways; they can be metabolized within mitochondria or be transported to the endoplasmic reticulum, where several enzymes, including GPAT, AGPAT, Lipin, and DGAT, catalyze the formation of triglycerides (TGs) [5,6,7]. The resulting TGs bind to the microsomal triglyceride transport protein (MTTP), which assists in the generation of chylomicrons in the endoplasmic reticulum [8,9]. Depending on the cellular lipid load, TGs can also be temporarily stored in cytosolic lipid droplets (CLDs) within enterocytes [10], from which they can be released by lipases, such as ATGL and HSL, and further trafficked to chylomicrons [11,12]. Afterwards, chylomicrons are transported to the Golgi complex and are secreted from enterocytes to the lymph [2]. As long chain FAs (LCFAs) go through these complex absorption, rebuilding, and secretion steps, medium chain fatty acids (MCFAs) are processed faster and more easily. Given their lower mass, MCFAs are hydrolyzed rapidly and more completely by pancreatic lipase than LCFAs, and do not form micelles. In addition, their short carbon chain makes them weak electrolytes that are highly ionized at neutral pH, which increases their solubility and accelerates their transporter-free absorption. Due to the bias of TG-assembling enzymes in enterocytes towards FAs with chains >12 carbons, MCFAs are not incorporated into TGs. Therefore, 95% of MCFAs are not integrated into chylomicrons, but are directly shed into the portal vein and travel quickly to the liver as FFAs. Therefore, MCFAs reach this organ much faster than LCFAs [13,14].

Canola oil is much appreciated by nutritionists due to its high unsaturated FA content. The oil is composed of 71% monounsaturated fatty acids (MUFAs), 21% polyunsaturated fatty acids (PUFAs), and only 6.3% saturated FAs [15]. Because of its plant sterol and tocopherol content, canola oil is thought to be cardioprotective [16], and canola oil-based diets reduce plasma TG and low-density lipoprotein cholesterol (LDL-C) levels, as well as biomarkers of coronary heart disease [17]. In contrast, coconut oil consists mainly of saturated FAs (92%) with a high lauric acid content (47%), and also other MCFAs (17%) [13]. Lauric acid with its 12-carbon atom chain, shares only some of the properties of MCFAs; however, during the digestion process, it can be released faster and absorbed more rapidly than LCFAs [13]. As a plant-derived oil, coconut oil is considered as a healthier alternative to animal fat, but it increases total cholesterol, high-density lipoprotein cholesterol (HDL-C), and LDL-C levels in the blood [13].

PPARs form a subfamily of the nuclear receptor family, which consists of PPARα, PPARβ/δ, and PPARγ [18]. They are expressed in various tissues at varying levels, and the individual roles of these receptors remain distinct. In the gastrointestinal tract, PPARα regulates the expression of genes that are associated with FA, cholesterol, glucose, and amino acid metabolism, transport, and intestinal motility in response to dietary lipids [19,20]. PPARβ/δ in the intestine regulates multiple processes, including cell proliferation, differentiation [21], and lipid uptake [22]. Compared to the small intestine, PPARγ is expressed at higher levels in the colon, where it inhibits dysbiotic Enterobacteriaceae expansion [23]. However, in both of these sections of the intestine, PPARγ is present at relatively higher levels in the proximal regions and its expression decreases towards the distal regions [24,25,26,27]. In the small intestine, PPARγ is directly exposed to ligands that naturally occur in food, with its activity being regulated by FAs, glutamine, curcumin, capsaicin, and vitamin E [28,29]. Thus, dietary composition impacts PPARγ functions. Though much attention has been paid to the anti-inflammatory [30,31,32,33,34,35,36,37] and anticarcinogenic role of PPARγ in the colon [38,39,40,41], and intestines in general [42], very little is known about its function in the small intestine. In this section of the intestine, its expression and nuclear translocation is activated during inflammation and injury [43]. We previously reported that intestinal PPARγ regulates adipocyte energy mobilization via the sympathetic nervous system during caloric restriction (CR) [44].

When considering that PPARγ is under-investigated in the small intestine and its importance in lipid metabolism in other tissues [45], we assessed its role in small intestine lipid metabolism using intestinal epithelium-specific PPARγ knockout mice. This approach is superior to antagonist treatment, which does not allow for tissue-specific inhibition of receptor activity. Here, we present evidence that PPARγ is preferentially involved in the metabolism of LCFAs in the small intestine.

## 2. Results

### 2.1. PPARγ Regulates Lipid Transit but Not Uptake in Small Intestine

**Figure 1:**
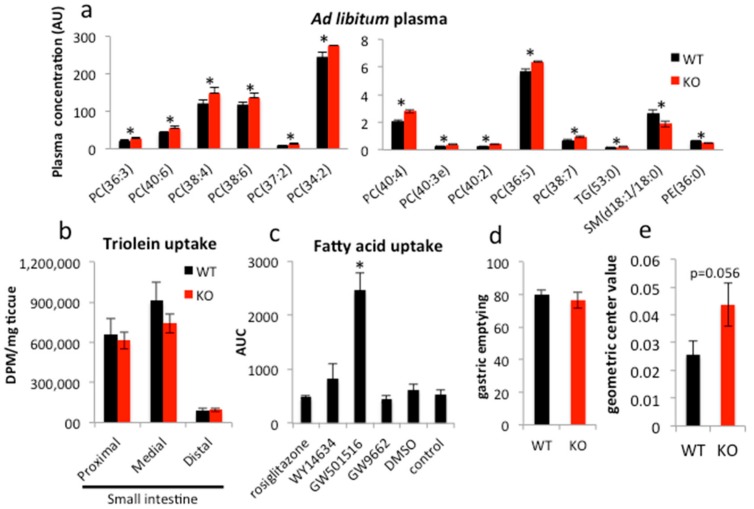
PPARγ does not affect lipid uptake, but regulates intestinal transit. (**a**) Blood was collected from mice fed ad libitum and plasma lipid composition analyzed (*n* = 5). (**b**) Lipid uptake was quantified by recording the radioactive tracer uptake (^3^H-triolein) in the duodenal epithelium 30 min after labeled oil gavage (wild-type (WT) *n* = 8, KO *n* = 12). (**c**) Following 24 h incubation with the indicated compounds, fluorescent fatty acids (FAs) were added to each well and uptake by Caco-2 cells measured over 2 h (*n* = 3). (**d**) WT and iePPARγKO mice were gavaged with FITC-dextran in canola oil and the fluorescence in the stomach and (**e**) small intestine measured. For the intestine, the geometric center was quantified 30 min after gavage (WT *n* = 12, KO *n* = 10). The Student’s *t*-test was performed for (**a**,**b**,**d**,**e**). For (**c**), one-way ANOVA with a Bonferroni post-hoc test was applied. Data are presented as means ± SEM (standard error of mean). * *p* < 0.05.

As we reported previously, the intestinal epithelium-specific PPARγ knockout mouse (PPARγ^Δ/Δ^ VillinCre^+/−^, or iePPARγKO) does not demonstrate any easily apparent phenotype in basic ad libitum conditions with respect to body weight, internal organ size, or plasma markers (TGs, FFAs, glucose, and cholesterol) [44]. However, advanced lipidomics analysis showed that plasma levels of several lipids differ in ad libitum iePPARγKO compared to their wild-type (PPARγ^fl/fl^VillinCre^−/−^, WT) littermates. Sphingomyelins (SMs, d18:1/18:0) and phosphatidylethanolamines (PEs, 36:0) were underrepresented in plasma from iePPARγKO as compared to WT mice (Figure 1a), whereas the concentrations of TGs (53:0) and several types of phosphatidylcholines (PCs) were higher in plasma from iePPARγKO mice than WT mice. In general, saturated FAs containing lipids were less abundant and unsaturated FAs occurred at higher concentrations in iePPARγKO when compared to WT mice. Choline is an essential component of PC; it is also essential for bile acid homeostasis and plays a role in the lipid uptake process. These observations suggested the involvement of PPARγ from the intestinal epithelium in lipid uptake and/or metabolism in this tissue.

To characterize the role of the intestinal PPARγ, we performed a lipid uptake test in the small intestine in iePPARγKO mice. In animals that were gavaged with a mix of canola oil and ^3^H-triolein, the amount of ^3^H-tracer taken up by the intestinal epithelium within 30 min after gavage did not differ between iePPARγKO and WT mice (Figure 1b). In analogous in vitro experiments, Caco-2 cells were treated with various agonists and antagonists of the different PPAR isotypes and were incubated with fluorescently labeled FAs. Only the agonist specific for PPARβ/δ, GW501516, clearly increased FA uptake (Figure 1c). In contrast, rosiglitazone, an agonist of PPARγ, and WY14634, a strong agonist of PPARα, which also weakly activates PPARβ/δ and PPARγ, did not significantly affect FA uptake. Furthermore, GW9662, an antagonist of all three PPAR isotypes, had no significant effect on FA uptake.

Next, we performed a gastrointestinal transit assay using fluorescently labeled FAs. Stomach emptying activity was not affected by the absence of PPARγ (Figure 1d). However, the assay revealed an increased intestinal transit speed in iePPARγKO compared to WT mice (Figure 1e). We conclude that although PPARγ does not affect lipid uptake in the intestinal epithelium, it regulates intestinal transit.

### 2.2. Long-Term Canola Oil Diet Results in Modest Body Composition Changes in iePPARγKO Compared to WT Mice

**Figure 2:**
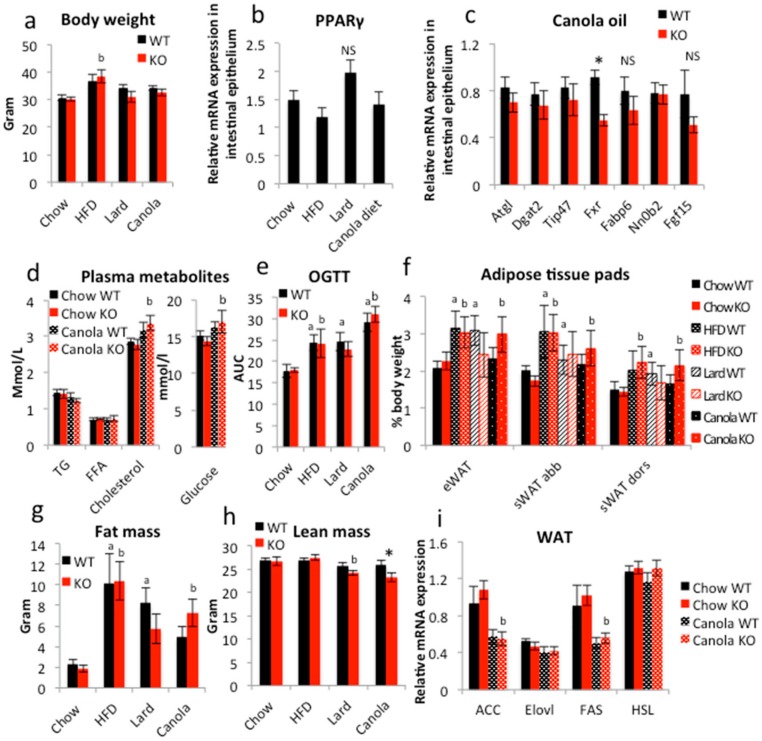
Long-term exposure to canola oil triggers mild body composition changes in iePPARγKO vs. WT mice. (**a**) Body weight of mice fed chow, high fat diet (HFD), lard diet, or canola oil diet (*n* = 7–10). (**b**) The relative mRNA expression levels of PPARγ and (**c**) lipid metabolism-associated genes in the duodenal epithelium were assayed by RT-qPCR (*n* = 9–18). (**d**) Concentration of TGs, FFAs, cholesterol, and glucose in plasma of mice fed chow and canola diet (*n* = 7–12). (**e**) Mice were submitted to oral glucose tolerance test (OGTT) and their plasma glucose levels monitored over 2 h (*n* = 5–9). (**f**) Weight of epididymal white adipose tissue (eWAT), subcutaneous abdominal WAT (sWAT abb), and subcutaneous dorsal WAT (sWAT dors) presented as % of total body weight (*n* = 8–10). (**g**) Total body fat and (**h**) lean mass were estimated using EchoMRI (*n* = 8–10). (**i**) Relative mRNA expression levels in epididymal WAT from chow and canola fed mice were measured using RT-qPCR (*n* = 8–10). One-way ANOVA followed by the Bonferroni post-hoc test was used to compare the experimental groups in (**b**,**d**,**f**,**i**). The two-tail Student’s *t*-test was applied to verify significance (*p* < 0.05) in (**a**,**c**,**e**,**g**,**h**). * *p* < 0.05 for canola iePPARγKO vs. canolaWT, ^a^ significantly differ from Chow WT, ^b^ significantly differ from Chow KO. Data are presented as mean ± SEM.

In order to disclose an iePPARγKO phenotype, we submitted the iePPARγKO mice to an 18-week feeding with different high-lipid diets. The animals were fed a standard high-fat diet (HFD; 60% energy from mixed fat sources) and two fat-rich diets with 45% energy from lard or canola oil. The latter two were set at 45% energy from fat because it was the maximum possible percentage at which the use of liquid canola oil still allowed for the production of solid food pellets. Control groups were fed standard chow containing 4.5% energy from fat, mostly of soy and sunflower origin. The samples were collected in the late morning following a 2 h fast during the resting/non-eating phase to avoid acute fat/oil effects as studied below. The animals fed HFD presented with the highest body weight gain, followed by the canola and lard diet groups when compared to the control animals (Figure 2a). However, only the weight increase of iePPARγKO mice fed the HFD was significant. All mice fed fat diets (HFD, lard, and canola) consumed less food than the control mice (Figure S1a). Interestingly, there were no significant differences in final body weight and food intake between iePPARγKO and WT mice in any of the four groups (Figure 2a and Figure S1a). Mice fed a HFD or lard diet presented elevated VO_2_ when compared to control mice (Figure S1b). Canola oil-fed mice exhibited a similar trend, but did not reach significance. Respiratory exchange ratio (RER) was reduced in all of the mice that were consuming fat diets (Figure S1c). No differences were noted for VO_2_, VCO_2_, RER, or heat release between iePPARγKO and WT mice (Figure S1b–e).

Gene expression analysis revealed that PPARγ expression in the intestinal epithelium of WT mice was not significantly modified by the fatty diets as compared to the control diet (Figure 2b). Among the genes whose expression in the intestine was affected by canola oil gavage, only *Fxr* was downregulated in iePPARγKO compared to WT mice after the 18-week canola oil diet (Figure 2c). Further perusal of the expression of FXR target genes, such as *Fabp6*, *Nr0b2*, and *Fgf15*, did not reveal significant changes, not least because of relatively high variability in expression. A trend of downregulation in the iePPARγKO epithelium was observed for the PPAR-regulated genes *Atgl*, *Dgat*2, and *Tip47*. Furthermore, canola oil diet did not trigger differences between iePPARγKO and WT mice in regards to plasma TG, FFA, cholesterol, and glucose levels (Figure 2d). In the oral glucose tolerance test (OGTT), mice that were fed fatty diets had significantly higher glucose plasma levels than control mice (Figure 2e and Figure S1f). Interestingly, mice that were fed a canola diet had higher glucose levels than HFD-fed mice. No differences in plasma glucose levels were found between iePPARγKO and WT mice at any of the time points of the OGTT for any diet. Similarly, liver size was comparable between the two genotypes (Figure S1g).

As expected, the mice that were fed fat diets had increased relative epididymal, subcutaneous abdominal, and dorsal white adipose tissue (WAT) weight when compared to control mice, with HFD mice having the highest amount of WAT (Figure 2f) and the canola oil diet-fed WT mice the lowest. The iePPARγKO mice fed a canola diet exhibited a trend towards heavier fat pads for all three of the diets tested and increased total body fat mass compared to their WT littermates, but the difference was not significant. EchoMRI confirmed the trend towards increased total body fat mass in canola oil-fed animals (Figure 2g) and revealed a significant decrease in the lean mass of iePPARγKO vs. WT canola diet mice (Figure 2h). Expression of *Acc* and *Fas* in the WAT of mice consuming the canola diet was decreased as compared to control mice (Figure 2i), but no difference was detected between iePPARγKO and WT mice. Thus, a canola oil diet increased the fat mass in iePPARγKO mice when compared to WT mice and resulted in a difference in body mass composition between the two genotypes. Furthermore, the effect on gene expression in the duodenum of these animals fed canola oil for 18 weeks was less than that observed with acute canola oil gavage (see below).

### 2.3. PPARγ Affects Lipid Metabolism in Duodenal Enterocytes

**Figure 3:**
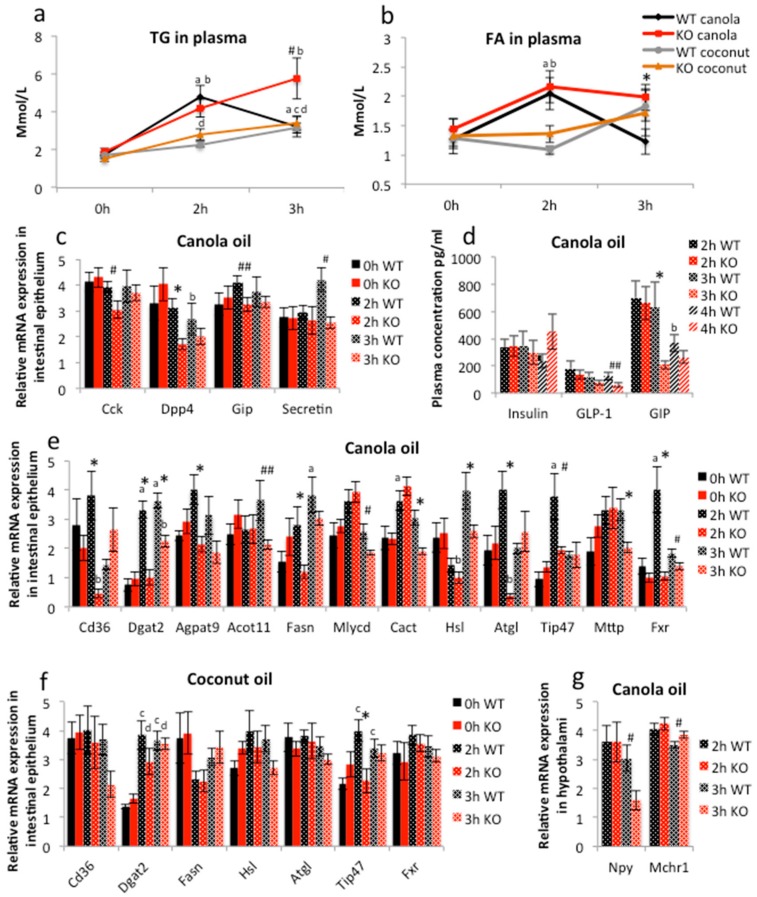
Canola oil gavage triggers differences in lipid metabolism signaling between iePPARγKO and WT mice. (**a**) Triglyceride (TG) and (**b**) free fatty acid (FFA) levels were measured in plasma after canola (*n* = 6) and coconut (*n* = 5–6) oil gavage. (**c**) Applying RT-qPCR, the relative mRNA expression levels in the duodenal epithelium were analyzed for intestinal hormones. (**d**) Plasma concentrations of insulin, GLP-1, and GIP were measured for WT and iePPARγKO mice gavaged with canola oil (*n* = 6–7). (**e**) The relative mRNA expression levels were quantified for lipid metabolism-associated genes in the duodenal epithelium of animals gavaged with canola oil and (**f**) coconut oil (*n* = 5–6) and (**g**) for hunger-related genes in the hypothalami of canola oil gavaged WT and iePPARγKO mice (*n* = 6–10). * Significant differences between iePPARγKO and WT mice. ^#^ *p* < 0.05; ^##^ *p* < 0.08. ^a^ Significant differences between the labeled group and 0 h WT canola, ^b^ 0 h KO canola, ^c^ 0 h WT coconut, and ^d^ 0 h KO coconut. One-way ANOVA with a Bonferroni post-hoc test was applied for statistical analysis. Error bars depict the standard error.

As the long-term lipid challenge with canola oil disclosed a mild iePPARγKO phenotype, we challenged iePPARγKO and WT mice with acute lipid loads via a single gavage of canola oil (5 μL/g body weight), which is very rich in long chain unsaturated FAs, following an overnight fast. In WT animals, plasma TG and FFA levels were maximal after 2 h, with lower levels already at 3 h. In iePPARγKO mice, TG levels were still increased and FFAs remained higher at 3 h with significant differences from WT mice (Figure 3a,b). These results suggest that PPARγ in the intestinal epithelium impacts the processing of these molecules in the small intestine. WT and iePPARγKO mice that were gavaged with the same volume of coconut oil, which is very rich in saturated MCFAs, did not exhibit differences in plasma TGs or FFAs, which were highest at 3 h (Figure 3a,b), but TGs were significantly lower than after gavage with canola oil (Figure 3a). Plasma levels of total cholesterol, HDL, and glucose were similar in iePPARγKO and WT mice after either of the two oil gavages (Figure S2a,b). These results suggest that PPARγ selectively affected the intestinal processing of unsaturated LCFAs, but did not impact that of saturated MCFAs.

Because PPARγ is a transcription factor, we assessed whether the above observations result from changes in gene expression in the intestinal epithelium due to oil gavage, and whether tissue-specific deletion of PPARγ affects them. As PPARγ is expressed at higher levels in the proximal parts of the small intestine [24,27], we measured the mRNA levels in the duodenum. It noteworthy that the deletion of PPARγ did not significantly affect the expression of PPARα and PPARβ/δ, which could have impacted the results (Figure S2e). In WT mice, canola oil gavage stimulated the expression of *Gip* and *Secretin* after 2 and 3 h, respectively, whereas the expression of *Cck* (cholecystokinin) and *Dpp4* (dipeptidyl peptidase-4) was not affected (Figure 3c, Table S1). When compared to WT, *Cck*, *Dpp4*, and *Secretin*, expression was reduced in the iePPARγKO duodenum (Figure 3c, Table S1). Plasma gastric inhibitory polypeptide (GIP) protein levels were also significantly diminished in iePPARγKO when compared to WT mice at 3 h, and glucagon-like peptide-1 (GLP-1) at 4 h, after canola oil gavage (Figure 3d).

Canola oil gavage resulted in the stimulation of several genes of lipid metabolism in the duodenum of WT mice 2 and/or 3 h after gavage (Figure 3e and Figure S2c,e and Table S1). Importantly, the gene expression profiles differed between the WT and iePPARγKO duodenum (Figure 3e and Figure S2c,d, Table S1). In iePPARγKO mice, the genes stimulated in WT mice were expressed at lower levels 2 and/or 3 h after gavage (Figure 3e and Figure S2d, Table S1). Moreover, in iePPARγKO mice some of the genes were initially downregulated at 2 h compared to 0 h. Among the altered transcripts were those encoded by genes associated with lipid uptake (*Cd36*), TG synthesis (*Dgat2*, *Agpat9*), FA metabolism (*Acot11*, *Fasn*, *Mlysd*), FA transport to mitochondria (*Cact*), lipid droplet formation (*Hsl*, *Atgl*, *Tip47*), and chylomicron production (*Mttp*) (Figure 3e). Notably, the mRNA levels of *Fxr* were affected, suggesting a possible impact of PPARγ on bile acid signaling. Among the genes that were not influenced by the absence of intestinal PPARγ were those associated with cholesterol and lipid absorption (*Abca1*, *Abcg5*, *Ppap2a*), lipid metabolism (*Lcad*, *Cpt-1*), lipoprotein composition (*ApoAIV*, *ApoB*, *Vti1A*), mitochondrial ATP production (*Atp5e*), and mitochondrial respiratory chain (*Uqcr2*). Notably, the *Ppar*γ mRNA level was not altered in WT mice after canola oil gavage (Figure S2f). Importantly, the level of mRNA of *Ppar*α was downregulated in iePPARγKO 2 h after the gavage (Figure S2e). However, the expression pattern of *Ppar*α and *β*/*δ* did not differ between iePPARγKO and WT mice at any time point following the gavage (Figure S2e), which indicates that their action in lipid metabolism is independent of *Ppar*γ.

In contrast to the above results, coconut oil gavage did not trigger differences in intestinal epithelium gene expression between iePPARγKO and WT mice, with the exception of *Tip47*, which is involved in the biogenesis of lipid droplets (Figure 3f) and shares a significant homology with the other members of this family, including perilipin and adipophilin [46].

Interestingly, *Npy* (*p* = 0.03), which is associated with the regulation of metabolism and behavior, and *Mchr1* (*p* = 0.05) whose product is thought to have a number of functions, including the regulation of appetite [47,48], were down- and slightly up-regulated, respectively, in the hypothalami of iePPARγKO compared to WT mice 3 h after canola oil gavage (Figure 3g). Meanwhile, the expression of other hypothalamic appetite-related genes (*Hpmr*, *Hcrtr1*, *Mc4r*, *Npbw1*) was not affected in iePPARγKO mice after canola oil gavage (Figure S2g).

Collectively, these results show that PPARγ in enterocytes is activated by canola oil to specifically control pathways that are connected with FA metabolism and mitochondrial function, and possibly affect some hypothalamic functions. In contrast, PPARγ activity appears to not be influenced much by saturated MCFAs. This finding is in line with the previously reported preference of PPARγ for PUFAs as ligands [49].

## 3. Discussion

A previous investigation of iePPARγKO mice fed a chow diet when compared to WT mice did not reveal an easily recognized phenotype [44]. Here, a more in-depth plasma analysis revealed differences in circulating lipids, particularly the PC fraction. We also found that, after long-term exposure to a canola oil-rich diet (18 weeks), the iePPARγKO mice had reduced relative lean mass compared to WT animals, which correlated with a trend of higher fat mass, in line with the previously reported adipose tissue dysregulation in these animals under CR [44]. Furthermore, after canola oil gavage, we observed changes in plasma TG and FFA levels between iePPARγKO and WT mice. These modifications in circulating lipids were not due to faulty lipid uptake, but were correlated with increased intestinal transit in iePPARγKO mice, and, importantly, iePPARγ-dependent changes in enterocyte gene expression. The modulated genes are associated with lipid metabolism, mitochondrial functions, and gut hormones.

**Figure 4:**
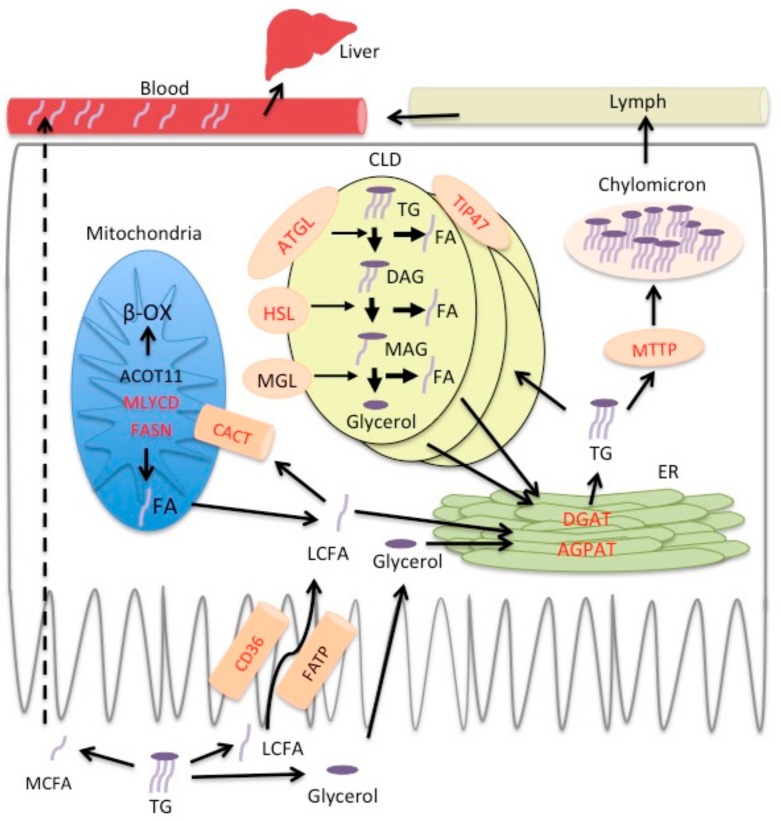
Model of lipid processing regulation by PPARγ in enterocytes. Red font indicates genes whose upregulation is lost or expression is reduced in enterocytes of iePPARγKO mice after canola oil gavage. Following intestinal digestion, FAs and glycerol are absorbed by enterocytes. Medium chain FAs (MCFA) travel through the enterocyte directly to blood (portal vein) (dashed arrow) and are transported to the liver as free FAs. Long chain FAs (LCFAs) are taken up by the enterocytes with the assistance of transporter proteins (CD36 and FATP). FAs are trafficked to mitochondria, where they are catabolized, or to the endoplasmic reticulum (ER), where there serve as substrates for TG assembly. Depending on the lipid load, TGs can be temporarily stored in cytoplasmic lipid droplets (CLD) or incorporated into chylomicrons and secreted into the lymph.

As mentioned above, the plasma levels of several PCs were increased in iePPARγKO mice. In humans, PCs are derived mostly from bile acids (10–20 g/day), but also from the diet (1–2 g/day) [2]. If this also prevails in rodents, the level of PCs in plasma may reflect changes in bile acid metabolism. The loss of *Fxr* upregulation after canola oil gavage and canola diet in iePPARγKO mice also suggests that bile acid metabolism may be affected by the absence of PPARγ in the intestinal epithelium. Although there was a trend for a lower expression of several FXR target genes in iePPARγKO mice, the difference from WT did not reach significance. Therefore, a possible alteration of the role of FXR in the iePPARγKO phenotype remains to be investigated more in-depth in the future. Choline and its metabolites are needed for the structural integrity of cell membranes and their signaling roles, cholinergic neurotransmission, and participation in the *S*-adenosylmethionine (SAMe) synthesis pathways. As PCs are the predominant type of phospholipids in the intestinal lumen and were increased in iePPARγKO mice, we evaluated whether intestinal lipid uptake was perturbed in iePPARγKO mice. Although iePPARγ did not modify the amount of lipid that was taken up, canola oil gavage led to differences in plasma lipid levels between WT and iePPARγKO mice, which is in line with alterations in epithelial gene expression in the latter. The persistence of high plasma TG and FFA levels 3 h after gavage may suggest a modified intestinal transit time, release from the epithelium, or clearance from the bloodstream. The expression of several genes in the intestinal epithelium was reduced in the absence of iePPARγ. Together, these genes are implicated in all of the processes of lipid metabolism in enterocytes (Figure 4), including lipid transport (*Cd36* [50,51,52,53]), lipolysis (*Hsl* [50,52,54,55], and *Atgl* [51,55,56]), and various lipid metabolism pathways (*Cact* [57], *Fasn* [58,59], *Mlycd* [60], *Dgat2* [50,52,55,59], and *Agpat9* [51,55,61]). Interestingly, *Acot 11* (hydrolysis of various coenzyme A esters), *Tip 47* (lipid droplet formation), and *Mttp* (chylomicron assembly) were previously not associated with PPARγ in any tissue. In addition, *Tip 47* was differentially expressed between the two phenotypes after both canola oil and coconut oil gavage, suggesting that coconut oil contains some FAs that may moderately affect some PPARγ pathways. In the future, an investigation at the protein level (expression, posttranslational modifications) will further the present study.

Previously, we demonstrated that the intestinal PPARγ negatively affects the expression of incretins and their plasma levels during CR [44]. Here we showed that, after canola oil gavage, the mRNA and plasma levels of incretins are reduced in iePPARγKO compared to WT mice, which demonstrates the different roles of PPARγ in intestinal hormone synthesis in different nutritional contexts. Interestingly, based on previously published findings by others [62,63], this downregulation of GIP, CCK, or secretin levels in iePPARγKO mice may explain the difference in intestinal passage time, which was increased in these mice. When considering that the lipid load increases gut motility [64], we hypothesize that fat may act through PPARγ to regulate intestinal transit, a function that has also been attributed to PPARα [19]. Such a putative role of PPARγ remains to be studied, as we have observed slightly accelerated transit in the iePPARγKO mice. Interestingly, gavage with saturated fat-rich coconut oil had much weaker effects than canola oil. This is very much in line with PPARγ having a preference for PUFAs as ligands, which are enriched in canola oil, over saturated FAs as ligands, which are abundant in coconut oil [28,29,49].

Interestingly, an 18-week-long canola oil feeding with sampling after 2 h fast during the resting non-feeding time did not produce the same clear effects as acute gavage. These observations suggest that the feeding time and the type of fats in the food directly regulate PPARγ activity in the intestinal epithelium. Alternatively, long-term fat feeding may change the lipid uptake and processing in the intestine [65], and, thus, the iePPARγKO phenotype may be attenuated under this condition. Nonetheless, we observed an effect of long-term canola oil feeding with a change in the ratio between lean and fat body mass in iePPARγKO mice when compared to WT mice. This difference in body composition may originate from a faulty metabolism of lipids in the intestine, as discussed above. Alternatively, canola-activated PPARγ could also lead to a similar effect on lipid release from WAT via PPARγ-dependent sympathetic nervous system signaling, as described previously [44]. The absence of this signal would result in fat retention in the adipose tissue, which is in line with our present observations.

Oils with different FA composition causing different phenotypes in iePPARγKO implies that intestinal PPARγ specifically regulates complex pathways under the influence of LCFAs, which are enriched in canola oil as naturally occurring agonists of PPARγ [28,29]. Our results suggest that consumption of oils rich in PPARγ agonists may improve the efficiency of lipid metabolism in the intestine and also impact the lean/fat mass ratio. In conclusion, we hypothesize that intestinal epithelium PPARγ affects lipid processing and/or the storage in enterocytes and adipose tissue, and its deletion would result in delayed trafficking in enterocytes and, possibly as described for CR [44], fat retention in adipose tissue.

## 4. Materials and Methods

### 4.1. Mouse Handling

All of the animal experiment protocols were approved by the Vaud Cantonal Authority (SCAV 24735; authorization: VD 2440.3; 01 April 2015), Switzerland. As described previously [44], the intestinal epithelium-specific PPARγ knockout mouse was obtained by crossing floxed *Pparγ* (PPARγ^fl/fl^) mice with mice expressing the Cre recombinase transgene under control of the villin promoter (VillinCre^+/−^). The offspring PPARγ^Δ/Δ^ VillinCre^+/−^ mice with targeted disruption of PPARγ in the intestinal epithelium were named iePPARγKO mice and were used in parallel with littermate control PPARγ^fl/fl^ (WT) mice with the same genetic background. Male mice were kept under a 12-h light/12-h dark cycle in standard housing cages. The animals were fed a standard laboratory diet, unless otherwise stated, and housed with free water access. For the oil gavage experiments, 10 to 12-week-old mice were fasted overnight. The next morning, the mice received 5 μL canola or coconut oil (Sigma-Aldrich, Buchs, Switzerland) per gram of body weight via gavage. The animals were dissected directly after overnight fasting or 2 and 3 h after oil gavage. The mice were euthanized using CO_2_ and blood was drawn by cardiac puncture. The blood was mixed with 2% aprotinin-EDTA (Sigma, Mendota Heights, MN, USA) and DPPIV inhibitor (Merck, Kenilworth, NJ, USA), centrifuged for 10 min at 8000× *g*, and plasma frozen. Epididymal WAT, subcutaneous abdominal WAT, dorsal WAT, and liver weight were recorded. Duodenum scrapings and hypothalami were collected. All tissues were frozen in liquid nitrogen and stored at −80 °C until use.

For the diet experiments, five-week-old mice were randomly assigned to one of the diets: chow containing 4.5% energy from fat, mostly of soy and sunflower origin (Diet 3436, Provimi Kliba AG, Penthalaz, Switzerland); HFD with 60% kcal from fat in which the main fat source was lard (D12492 OpenSource Diets, Research Diets, New Brunswick, NJ, USA); or, HFD with 45% kcal fat from canola oil or lard (custom made modified D12451 diets, Research Diets). Body weight and food intake were measured weekly. After 15 weeks feeding with these diets, metabolic parameters (VO_2_, VCO_2_, heat production) and locomotor activity were monitored for three days using the Comprehensive Lab Animal Monitoring System (CLAMS, Columbus Instruments, Columbus, OH, USA). After 16 weeks of feeding with the control and HFDs, mice were submitted to the OGTT. Briefly, mice were fasted overnight, placed in single cages, and the first blood samples drawn from the tail. Next, the mice were gavaged a glucose solution and received the equivalent of 3 mg of glucose per gram body weight. Blood glucose levels were monitored after 15, 30, 60, 90, and 120 min. After 17 weeks of the diet, bedding maintained in the cage for 24 h was collected. Feces were separated from the bedding, dried, and fecal energy load measured using direct calorimetry (IKA-Kalorimeter C2000; IKA^®^-Werke GmbH & Co. KG; Staufen, Germany). Afterwards, the mouse body composition was measured under anesthesia using an EchoMRI whole-body composition analyzer (EchoMRI, Huston, TX, USA). After the EchoMRI, the mice were given 1 week to recover and then dissected following the procedure described above between 9 a.m. and 11 a.m. following 2 h fasting.

### 4.2. Intestinal ^3^H-Triolein

After overnight fasting, mice received 200 μL canola oil containing 15 μCi 3H-triolein by gavage and sacrificed 30 min later. Blood was removed by perfusing the heart for 3 min with PBS. The intestinal lumina was flushed four times with 5 mM taurocholate, the small intestine divided into three equal segments (proximal, medial, and distal), and the segments were dissolved in Solvable^TM^ (Perkin Elmer, Courtaboeuf, Villejust, France) overnight at 60 °C and incubated in scintillation fluid (Betaplate Scint, Perkin Elmer, Waltham, MA, USA). The radioactivity in each intestinal segment was measured by a liquid scintillation analyzer.

### 4.3. Gastric Emptying and Intestinal Motility

Overnight-fasted mice were gavaged with 200 μL of 5 mmol/L FITC-dextran (70 kDa FITC-dextran, Sigma) in canola oil and sacrificed 30 min later. Animals’ small intestines were divided into 10 equal parts. The stomach and each part of the intestine was opened longitudinally, vortexed thoroughly with PBS, and centrifuged at 1200 rpm for 5 min. The intensity of fluorescence in the supernatant was measured. The geometric center used as an index of intestinal transit was calculated as the sum of the % fluorescence per segment × segment number [66].

### 4.4. RT-qPCR

RNA was isolated from intestinal scrapings using the RNeasy mini kit (Qiagen, Hombrechtikon, Switzerland). Samples were thawed in lysis buffer, disrupted using a syringe and needle, and processed following the manufacturer’s recommendations. RNA was extracted from adipose tissue and the hypothalamus using the RNeasy Lipid Tissue mini kit (Qiagen). SuperScript^®^ II Reverse Transcriptase (Thermo Fisher Scientific, Lausanne, Switzerland) and random primers (Promega, Madison, WI, USA) were used for the reverse transcription step for all of the samples. Quantitative real-time PCR (qRT-PCR) reactions were carried out using the Applied Biosystems 7900HT (Thermo Fisher Scientific) with the SYBR green PCR Master Mix (Applied Biosystems, Thermo Fisher Scientific). Primers used for qRT-PCR are listed in Table S2.

### 4.5. Plasma Analysis

Serum (10 μL) samples were diluted with 0.9% NaCl (10 μL) buffer. All of the samples were spiked with an internal standard (10 μL). Subsequently, the samples were extracted with chloroform: methanol (2:1) solvent (100 μL), homogenized with a glass rod (serum) at 4 °C by adding two zirconium oxide grinding balls, vortexed (1 min), incubated at room temperature (1 h), and centrifuged at 5590× *g* for 3 min. An aliquot of the separated lower phase (60 μL) was mixed with a labeled standard mixture (three stable isotope-labeled reference compounds; 10 μL) and 0.5–1.0 μL injection used for the analysis. The sample order for analysis was established by randomization. Lipid extracts were analyzed on a Q-ToF Premier mass spectrometer (Waters, Milford, MA, USA), and combined with an Acquity ultra performance liquid chromatograph (UPLC/MS).

Plasma glucose, lipid, and cholesterol levels were measured using a Hitachi chemistry analyzer (Roche Diagnostics, Basel, Switzerland), according to the manufacturer’s instructions.

Plasma insulin, GLP-1, and GIP concentrations were estimated using Bio-Plex^®^ (Luminex Corporation, Austin, TX, USA).

### 4.6. Cell Culture

Caco-2 cells were maintained in high glucose DMEM supplemented with 10% fetal bovine serum, 100 U/mL penicillin and 100 U/mL streptomycin (all from Sigma-Aldrich) in a humidified atmosphere of 5% CO_2_ at 37 °C. Cells were cultured for 10 days after reaching confluence. Rosiglitazone, WY14634, GW501516, and GW9662 (all from Sigma) were added to the culture at final concentrations of 10 μM for 24 h. Control cells received the DMSO (Sigma) vehicle or no treatment. Afterwards, BODIPY-labeled fatty acids (QBT Fatty Acid Uptake Assay Kit, Molecular Devices, Wokingham, Berkshire, UK) were added to the culture and fluorescence measured over 2 h. The results are presented as area under the curve.

## Supplementary Materials

Supplementary materials can be found at www.mdpi.com/1422-0067/18/12/2559/s1.
