# Supplementary material for: PPARγ Modulates Long Chain Fatty Acid Processing in the Intestinal Epithelium
Source: Int J Mol Sci. 2017 Nov 28;18(12):2559. doi: 10.3390/ijms18122559 (PMC5751162; doi:10.3390/ijms18122559)
Supplement: Supplementary file 1 [file ijms-18-02559-s001.pdf]

PPARγ Modulates Long Chain Fatty Acid Processing in the Intestinal Epithelium

Kalina Duszka [1,2,3], Matej Oresic [4], Cedric Le May [5], Jürgen König [3,6] and Walter Wahli [1,2,7,*]

1 Lee Kong Chian School of Medicine, Nanyang Technological University, 11 Mandalay Road, Singapore 308232, Singapore; Kalina.duszka@univie.ac.at
2 Center for Integrative Genomics, University of Lausanne, Génopode, CH-1015 Lausanne, Switzerland
3 Department of Nutritional Sciences, University of Vienna, Althanstrasse 14, 1090 Vienna, Austria; juergen.koenig@univie.ac.at
4 Turku Centre for Biotechnology, University of Turku and Åbo Akademi University, Tykisokatu 6, 20520 Turku, Finland; matej.oresic@utu.fi
5 Institut du Thorax, INSERM, CNRS, UNIV Nantes, 44007 Nantes, France; cedric.lemay@univ-nantes.fr
6 Vienna Metabolomics Center (VIME), University of Vienna, Althanstrasse 14, 1090 Vienna, Austria
7 ToxAlim, Research Center in Food Toxicology, National Institute for Agricultural Research (INRA), 180 Chemin de Tournefeuille, 31300 Toulouse, France
* Correspondence: Walter.Wahli@ntu.edu.sg; Tel.: +65-6592-3927 or +65-9026-6430

Table S1: Relative expression of mRNA transcripts assayed by RT-qPCR

		Canola oil		Coconut oil	
**Gene**	**Time point**	**WT**	**iePPARγKO**	**WT**	**iePPARγKO**
		**Relative mRNA expression (normalized to reference gene)**			
Hormones					
Cck	2h	3.94 ± 0.20	3.05 ± 0.34		
	3h	3.95 ± 0.63	3.71 ± 0.31		
Dpp4	2h	3.11 ± 0.37	1.71 ± 0.21		
	3h	2.68 ± 0.61	2.01 ± 0.30		
Gip	2h	4.09 ± 0.29	3.26 ± 0.27		
	3h	3.75 ± 0.57	3.35 ± 0.22		
Secretin	2h	2.95 ± 0.26	2.62 ± 0.53		
	3h	4.21± 0.45	2.56 ± 0.23		
**Lipid metabolism**					
Cd36	2h	3.81 ± 0.83	0.46 ± 0.15	4.04 ± 0.84	3.60 ± 0.91
	3h	1.44 ± 0.18	2.63 ± 0.74	3.69 ± 0.53	2.14 ± 0.44
Dgat2	2h	3.32 ± 0.29	1.02 ± 0.27	3.85 ± 0.51	2.93 ± 0.45
	3h	3.65 ± 0.25	2.26 ± 0.21	3.66 ± 0.33	3.55 ± 0.20
Agpat9	2h	4.01 ± 0.51	2.14 ± 0.27		
	3h	3.14 ± 0.66	1.87 ± 0.38		
Acot11	2h	2.64 ± 0.46	2.66 ± 0.50		
	3h	3.64 ± 0.69	2.13 ± 0.17		
Fasn	2h	2.79 ± 0.63	1.21 ± 0.24	2.31 ± 0.28	2.25 ± 0.38
	3h	3.82 ± 0.61	3.03 ± 0.25	3.08 ± 0.30	3.44 ± 0.56
Mlycd	2h	3.61 ± 0.39	3.93 ± 0.35		

	3h	2.58 ± 0.24	1.86 ± 0.09		
Cact	2h	3.63 ± 0.34	4.12 ± 0.32		
	3h	3.05 ± 0.27	1.91 ± 0.12		
Hsl	2h	1.43 ± 0.23	1.00 ± 0.20	3.99 ± 0.73	3.44 ± 0.55
	3h	3.96 ± 0.62	2.59 ± 0.21	3.72 ± 0.47	2.71 ± 0.24
Atgl	2h	4.00 ± 0.64	0.38 ± 0.09	3.81 ± 0.21	3.64 ± 0.61
	3h	2.00 ± 0.18	2.58 ± 0.69	3.46 ± 0.33	3.01 ± 0.17
Tip47	2h	3.77 ± 0.79	1.95 ± 0.12	4.00 ± 0.38	2.27 ± 0.42
	3h	1.79 ± 0.11	1.77 ± 0.44	3.39 ± 0.31	3.23 ± 0.28
Mttp	2h	3.31 ± 0.47	3.38 ± 0.72		
	3h	3.33 ± 0.37	2.03 ± 0.18		
Fxr	2h	4.02 ± 0.78	1.05 ± 0.13	3.86 ± 0.34	3.57 ± 0.28
	3h	1.84 ± 0.14	1.40 ± 0.12	3.45 ± 0.23	3.13 ± 0.23
**Hypothalamus**					
Npy	2h	3.61 ± 0.57	3.61 ± 0.69		
	3h	3.03 ± 0.45	1.59 ± 0.33		
Mchr1	2h	4.06 ± 0.22	4.24 ± 0.23		
	3h	3.51 ± 0.11	3.86 ± 0.12		

Table S2: RT-qPCR primers

	Forward	Reverse
Abca1	GCACTGAGGAAGATGCTGAAA	AGTTCCTGGAAGGTCTTGTTCAC
Abcg5	CAGCAGCGTGTTGTATTGGA	AGCCGCGCACAGCAATACC
Acot11	CAGCCAGCCGGCTCTGTCAC	CACCTGAGACGGGCCTCGGA
Agpat9	CACCTGGCTGACGCTGGTGG	GCTGACTCCTTGGGGGCTCCT
ApoAIV	ACAGTTTCAGAAGACGGATGTCA	CGTACTAGCATCCCCAAGTTTG
ApoB	CCCCGTGCAAGAACTGGCTGA	GGGGAGCATTGTTAGGTTGAGGGC
ATGL	CCTGCCTGGGTGATCTTGAG	CTTGGCAGGCATGGGACATA
Atp5e	CCGGCAGATGGCGTAACAG	ACACATTTGCCCGAAGTCCATTG
Cact	GGCAGACGAGCCGAAACCCA	TCCAGGGGGTGCCCCACAAA
CCK	CCAATTTTTCCTGCCCGCAT	AGAAGGAGCAGTCAAGCCAAA
CD36	TGATACTATGCCCGCCTCTCC	TTTCCCACACTCCTTTCTCCTCTA
FASN	CAGAAATCGCCTATGGTTGTTG	GCTCAGCTGTGTCTTGGATGC
FXR	ACCCCAGAGAAGAACCGAGT	ACTTCTGGGATGGTGGTCCT

GIP	AGAGAGAGGCCCGGGCTTTGG	TCACTGAGACCTGAGTCGGCAG
HSL	TCAGGGACAGAGGCAGAGGAC	TCCACTTAGTTCCAGGAAGGAGTTG
Mttp	CCCGGGAAGCAAGTGGCAGG	TGCTCCGCCAGAGAAGGGCA
NPY	CAGAAAACGCCCCCAGAACA	GGGGATGGATGAGATGAGATGA
Ppap2a	TGTTGCTGGCTGCCATGCCT	GCCAAGCCCCAGTATGGCGA
PPARa	TCCTCAGTCAGCTGCCCCGT	ACCCTGAGGCCTTGTCCCCAC
PPARg	AGACCCAGCTCTACAACAGGCC	CAGACTCGGCACTCAATGGCCA
TIP47	ATGGAATCCGTGAAACAGGGTGTG	TGAGAGGTCCTGGAAGGAGTGAAT
Vti1a	GAGGCTGGGTACCAGATAGCA	CGCTGTATCTTTTCTCTGTCATGA

Primers not listed were purchased from Qiagen.

Supplementary figure 1

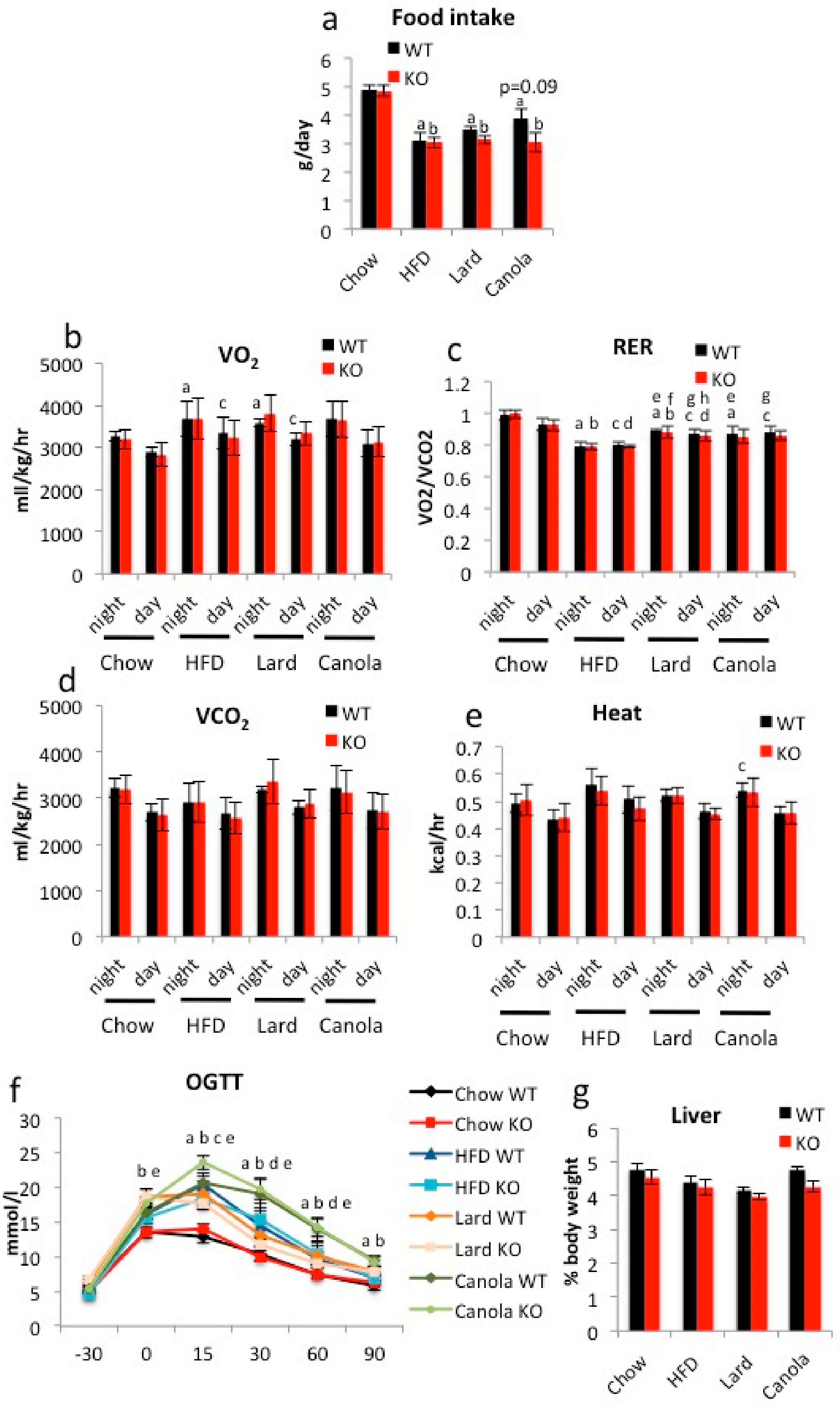

Figure S1. Parameters of fatty diet-fed iePPARγKO and WT mice.

(a) Food intake of mice fed different diets (n=7-10). [a]Significant difference between the labeled group and 0 h WT, [b]significant difference between the labeled group and 0 h KO. (b) Using indirect calorimetry, VO_2, (c) VCO_2, (d) RER, and (e) heat production during day and at night were assessed in mice (n=6-9). The symbols correspond to significant differences for the following data sets: [a]WT chow night; [b]KO chow night; [c]WT chow day;

[d]KO chow day; [e]WT HFD night; [f]KO HFD night; [g]WT HFD day; [h]KO HFD day. (f) The plasma glucose level was monitored over 2 hours (n=5-9) following glucose gavage. (g) The liver weight was recorded and presented as % of total body weight (n=7-10). One-way ANOVA followed by the Bonferroni post-hoc test was used to compare the experimental groups. All data are presented as mean ± SEM.

Supplementary figure 2

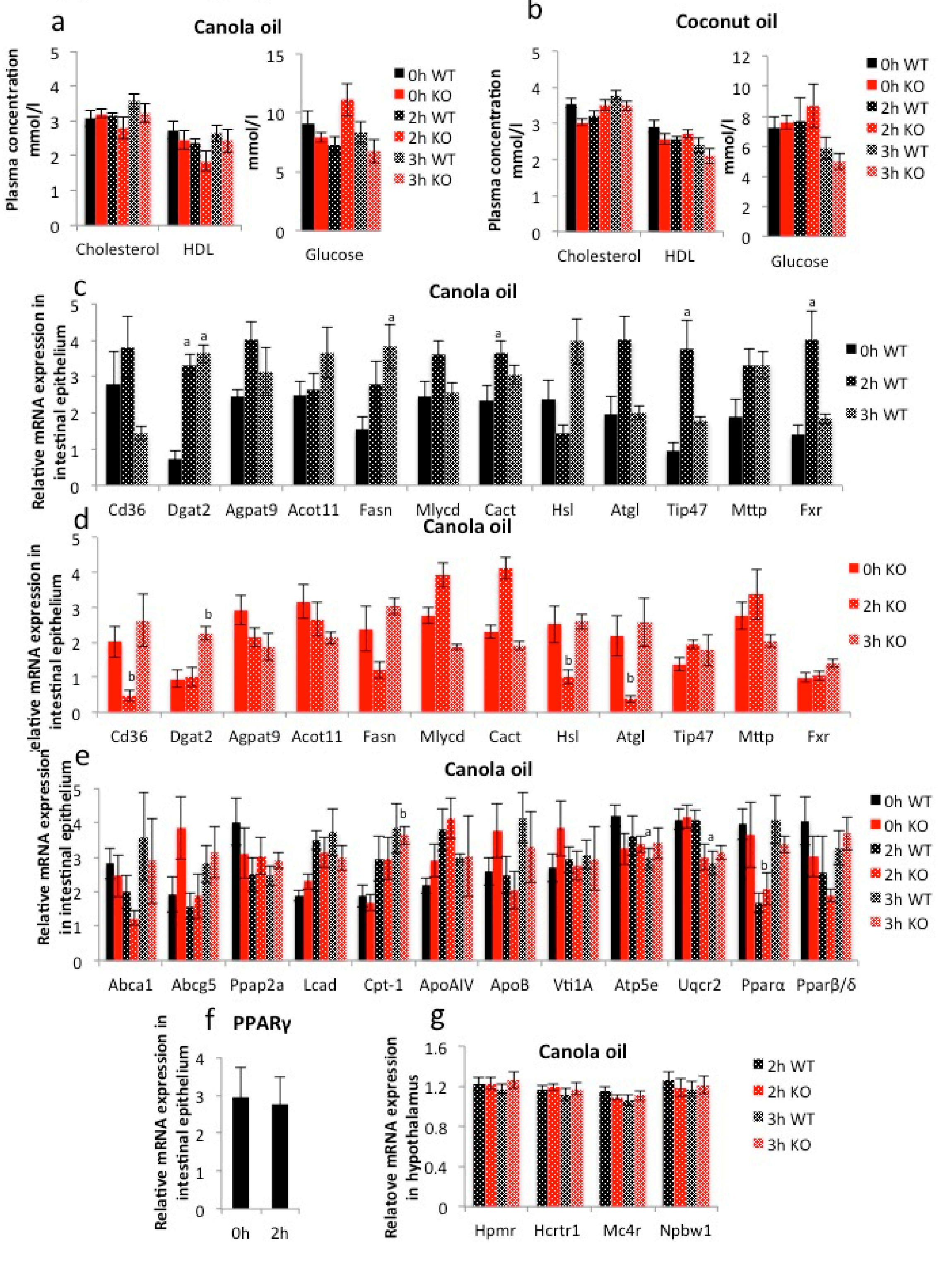

Figure S2. Plasma, intestine, and hypothalamus properties of iePPARγKO and WT mice gavaged with oil. (a) The concentrations of cholesterol, high-density lipoprotein (HDL), and glucose were measured in the plasma of mice gavaged with canola oil (n=6) and (b) coconut oil (n=5-6). (c, d, e, f) The relative mRNA expression levels in the intestinal epithelium (n=5-6) and (g) hypothalamus (n=6-10) were assayed by RT-qPCR. WT mice (c) are compared to iePPARγKO mice (d) for the same set of genes. The merge of the two graphs is presented in Figure 1f. ^{a}Significant difference between the labeled group and 0 h WT, ^{b}significant difference between the labeled group and 0 h KO. The data were analyzed using one-way ANOVA followed by the Bonferroni post-hoc test. Data are presented as mean ± SEM.
